# Implications of Patient-Reported Outcome Measures among patients with recently diagnosed type 2 diabetes

**DOI:** 10.1186/s13584-024-00592-1

**Published:** 2024-01-31

**Authors:** Nura Abdel-Rahman, Orly Manor, Einat Elran, David Siscovick, Ronit Calderon-Margalit

**Affiliations:** 1 Braun School of Public Health, The Hebrew University of Jerusalem Hadassah Medical School, 91120 Jerusalem, Israel; 2 Maccabi Healthcare Services, Tel Aviv, Israel; 3 The New York Academy of Medicine, New York, USA

**Keywords:** Type 2 diabetes, Patient-Reported Outcome Measures, Quality indicators, Quality of diabetes care

## Abstract

**Background:**

For the past two decades, the assessment of the quality of diabetes care has mostly relied on clinical quality indicators. These have not included Patient-Reported Outcome Measures (PROMs) which provide information on outcomes deemed valuable by patients. We aimed to examine the potential utility of PROMs in type 2 diabetes care and to study the association of PROMs with patients' characteristics and clinical quality indicators.

**Methods:**

A cross-sectional survey of recently (≤ 4 years) diagnosed patients with type 2 diabetes (n = 392) in the setting of a large health plan. PROMs were based on two well-validated questionnaires, the Problem Areas in Diabetes (PAID) one-page questionnaire that measures diabetes-related distress, and the ten item PROMIS-10 global health questionnaire that measures general health. Additional items were added following a previous qualitative study among Israeli patients with diabetes. The survey was carried out using phone interviews, and data collected were linked to the electronic medical records. Multivariable regression models were used to assess the associations of socio-demographic variables and clinical quality indicators with the PROMs.

**Results:**

About a fifth of participants (22%) had high diabetes-related distress (PAID score ≥ 40), a third reported that they did not feel confident in self-management of diabetes and about a third reported having sexual dysfunction. Women, younger patients, and those with a low education level (≤ 12 years) reported worse general health, were more likely to experience high diabetes-related distress, and to have low confidence in diabetes self-management. Interestingly, performance of all seven diabetes quality indicators was associated with worse general health and high diabetes-related distress. Of note, levels of glycated hemoglobin, LDL-cholesterol, or blood pressure were not associated with PROMs.

**Conclusions:**

PROMs provide important information on patient self-reported health status and are likely to reflect aspects of the quality of care that are not otherwise available to clinicians. Thus, the use of PROMs has the potential to expand the evaluation of diabetes care and promote patient-centered care. We recommend that policy-makers in the Ministry of Health and health maintenance organizations implement PROMs for assessing and improving the care for patients with type 2 diabetes.

**Supplementary Information:**

The online version contains supplementary material available at 10.1186/s13584-024-00592-1.

## Introduction

Healthcare has become more patient-centered in recent decades, with the measurement of quality of care gaining increasing attention [1, 2]. Evaluating quality of primary care based on clinical indicators, while highly important [3], may not capture all relevant outcomes of care [4]. Since the 1980s, patient reports are recognized as critical to evaluating quality of care in healthcare services [5–8]. Following a series of articles in the New England Journal of Medicine that called for a transformation in healthcare measurements [1, 2, 9], Patient-Reported Outcome Measures (PROMs) have evolved rapidly in the past decade. Patient-Reported Outcomes are defined as “any reports coming directly from patients about how they function or feel in relation to a health condition and its therapy”[10]. PROMs are usually collected using two types of questionnaires, generic and disease-specific [11]. PROMs have been used in a variety of settings including acute illnesses (e.g., Acute Myocardial Infarction), procedures (e.g., hip replacements), and in the field of oncology [12–14]. In these clinical settings, responding to the collected PROMs was found to be associated with important clinical outcomes, such as improved symptoms management, enhanced psychological well-being, and longer survival [12–14].

Diabetes care aims to prevent complications and to maintain satisfactory quality of life [15]. Thus, the American Diabetes Association (ADA), recommends routine monitoring of clinical indicators and PROMs [15, 16]. However, the utilization of PROMs in type 2 diabetes care poses challenges, including the unclear time of onset of the disease since type 2 diabetes can be undetected for years. Additionally, diabetes is often accompanied with many comorbidities making it difficult for the patient to distinguish the effects of the disease and its treatment from other comorbidities. A recent review concluded that adoption of PROMs in type 2 diabetes care remains uncommon and non-routine [11]. Indeed, only a few countries have considered PROMs for routine measurement of diabetes quality care [17–19] and there are no PROMs for diabetes that are widely used. Recently, the International Consortium for Health Outcomes Measurement (ICHOM) recommended a standard set of PROMs for diabetes [20]. Yet, national programs primarily have been using clinical quality indicators for evaluating quality of type 2 diabetes care [21–24]. Studies have shown controversial results regarding the associations between PROMs and clinical quality indicators, varying from significant associations (e.g. adequate control of HbA1c was associated with better physical and mental health [25]) to weak or non-significant associations [26, 27], and more research is needed.

The current study is part of a larger study that was conducted in the framework of the Israeli National Program for Quality Indicators in Community Healthcare which aimed to incorporate PROMs for diabetes within the Israeli national quality indicator set for diabetes. Since 2002, the Quality Indicators in Community Healthcare program has measured quality of community healthcare provided to all Israeli patients with indicators measuring mostly process of care. The assessment of quality indicators for diabetes constitute the flagship of the program [24, 28], with 11 indicators dedicated to diabetes care. We aimed to examine the potential utility of PROMs in type 2 diabetes care and to study the association of PROMs with patients' characteristics and clinical quality indicators.

## Methods

### Study design and setting

We conducted a cross-sectional study of recently (≤ 4 years) diagnosed patients with type 2 diabetes. We chose to focus on recently diagnosed patients as the first step in future routine use of PROMs. The study was conducted in the setting of Maccabi Healthcare Services, Israel's second largest health plan that provides community healthcare to 27% (2.4 Million) Israeli residents [29]. Phone interviews were conducted using structured questionnaires by trained interviewers, between March and July 2019. Each interview required about 10 min to complete.

### Study sample

Diabetes was ascertained between June 1, 2015 and December 31, 2016 (i.e. diabetes duration ≤ 4 years in 2019) based on one or more of the following criteria (according to the Israeli national definition of diabetes): (a) purchase of at least three prescriptions of anti-diabetic medications in 3 different months, (b) two random glucose measurements ≥ 200 mg% conducted at least 30 days apart, or (c) HbA1c ≥ 6.5% measured at least once. We included patients with diabetes aged 45–85 years who were fluent in Hebrew or Arabic. Potential participants were randomly sampled from the population of patients with diabetes using a stratified random sample. Strata were defined by ethnicity and age groups to ensure a representative sample for those who met the inclusion criteria. Of the 807 who were contacted, 392 agreed to participate in the survey (response rate; 48.6%). Participants and non-responders were similar with respect to age (60.5 ± 8.1 vs. 61.9 ± 8.5 years, respectively), socio-economic position score (5.8 ± 2.2 vs. 6.3 ± 2.0, respectively) and quality indicators achievement. The proportion of women among responding participants was non-significantly higher compared with non-responders (41.3% vs. 34.9%, respectively; p = 0.07).

### Socio-demographic variables and covariates

Socio-demographic data included gender (female vs. male), age in 2019 (categorized: 48.0–55.0, 55.1–64.9, and 65.0–84.0, based on the year of birth), and ethnicity (Jewish vs. Arab, based on participant’s spoken language). Socio-economic position was defined on the basis of the residential address, using scores ranging from 1 (low) to 10 (high). The scores are allocated to small statistical areas by the Israeli Central Bureau of Statistics [30] and updated by the POINTS Location Intelligence Company using current sociodemographic and commercial data [31]. These variables were retrieved from the electronic medical records.

Also, the data included marital status (married/cohabited vs. others: single, divorced, or widowed), country of birth (categorized: Israel vs. others), years of education (categorized: < 12, 12, and > 12 years), smoking (never, ever and current smokers), religion (secular, traditional, religious and orthodox), and diabetes duration (number of years with diabetes), these variables were patient-reported. Missing data of education (8.7%) were imputed using multiple imputations by chained equations (MICE) using age, gender, ethnicity, and socio-economic position.

### Quality indicators and clinical data

Data on seven process indicators and three intermediate-outcome indicators were collected according to the Israeli national quality indicator set [32]. Process indicators included measurement of HbA1c, LDL-cholesterol, blood pressure, urinary protein, serum creatinine, ophthalmological visit, and administration of influenza vaccine. Attainment of each indicator was defined as performance at least once in the year following diabetes diagnosis (2017). In addition, a composite score for all process indicators was calculated, indicating the total number of performed process indicators in 2017, categorized as (0–4, 5, 6, or 7).

Intermediate-outcome indicators assessed whether patients achieved adequate control (adequate vs. poor control), using the last measurement in 2017. Adequate control of glycemic control was an age-specific target (HbA1c ≤ 7% for patients aged ≤ 74 years and HbA1c ≤ 8% for patients aged ≥ 75 years) [33]. Adequate control of blood pressure was defined as systolic blood pressure ≤ 140 mmHg and diastolic blood pressure ≤ 90 mmHg. For LDL-cholesterol, control was defined as a level ≤ 100 mg/dl [33].

Comorbidities (presence vs. absence), based on diagnosis and procedures, included cardiac disease (ischemic heart disease or heart failure), retinopathy, visual loss, end stage renal disease, or lower limb amputation.

Quality indicators and clinical data were retrieved from the electronic medical records.

### Questionnaire construction and PROMs

The questionnaire construction was based on incorporating both the standard set recommended by ICHOM [20] and the results of our previous qualitative study that identified valuable aspects for patients with type 2 diabetes [34]. We adopted the Problem Areas in Diabetes (PAID) [35] questionnaire as the diabetes-specific questionnaire. PAID covered most of the domains that arose in our previous qualitative study and ICHOM recommended using PAID as the standardized diabetes-specific tool for PROMs [20]. PAID evaluates diabetes-related distress, i.e. patient's worries, fears, and burdens related to diabetes [35, 36]. The answers of the 20-items of PAID were summed and multiplied by 1.25 to generate a total score ranging from 0 to 100. And then dichotomized (PAID ≥ 40 vs. < 40), a score ≥ 40 indicates a high diabetes-related distress [37].

We selected the Patient-Reported Outcomes Measurement Information System (PROMIS-10) [38] as the generic tool for measuring general health, in line with other Israeli program that has been collecting PROMs for other diseases [39]. The PROMIS-10 questionnaire included 4-items that were summed to generate the global physical health (GPH) score and 4-items that generated the global mental health (GMH) score. These scores were transformed to T-score distributions, where higher scores indicate better health. Also the PROMIS-10 questionnaire included two items; the one for rating general health and the second for rating social activity [40].

According to our qualitative study [34], there were aspects deemed valuable to patients with diabetes that were not covered in the above-mentioned questionnaires. To get a full scope of these aspects, 3-items were added from the Diabetes Distress Scale [41] (confident in ability to manage diabetes, doctor doesn’t give clear enough directions and doctor doesn’t take my concerns seriously) and 4-items were added by the authors (medication costs, shared decision-making, sexual dysfunction and integrated care under one roof). All questions referred to whether the issue was a problem, with answers rated on a 5-point Likert scale, and then dichotomized (yes vs. no problem); the answers "serious problem", "somewhat serious" and "moderate problem" were classified as "yes" whereas, "no", "minor" and "irrelevant" were classified as "no".

Finally, the questionnaire collected sociodemographic data, information on smoking, and duration of diabetes. The questionnaire was translated to Hebrew and Arabic and back-translated. The Cronbach’s α-coefficient of PAID and PROMIS-10 indicated satisfactory reliability (0.92 and 0.84, respectively).

### Statistical analysis

Multivariable regression models were constructed to assess the associations between socio-demographic and quality indicators with PROMs scores. The following independent variables were studied: gender, age, ethnicity, level of education, presence of comorbidities, adherence to each clinical quality indicator (11 indicators including the process composite score), and the continuous value of HbA1c, blood pressure, and LDL-cholesterol.

Binary and multinomial logistic regressions were constructed to explore the associations between the independent variables and the categorical dependent variables; PAID and the additional items. Whereas generalized linear models were used to explore the associations between the independent variables and the continuous dependent variables; GPH and GMH. Model 1 included the socio-demographic variables and Model 2 included the socio-demographic variables and the clinical variables. Variables included in the models if they had a significant association with one of the dependent variables in the univariate analyses.

Statistical analysis was performed with IBM SPSS Statistics for Windows, Version 25.0 (Armonk, NY: IBM Corp). Two-sided p value < 0.05 was considered to be statistically significant. Regression coefficients β and odds ratios (ORs) along with 95% confidence intervals (CIs) were reported.

Ethical approval was obtained from the Institutional Review Board of Maccabi Healthcare Services.

## Results

Table 1 presents the characteristics of the study population. Mean age of participants was 60.5 years (SD:8.1) at the time of the interview, 41.3% were women, 18.9% were Arab, and 45.7% with more than 12 years of education. Almost 30% had at least one comorbidity, 73.0% were treated with oral anti-diabetic medications (none was treated with insulin), and 64.2% performed at least six of the seven process indicators in 2017.

**Table 1:** Socio-demographic and clinical characteristics of the study population (n = 392)

Variable	Percent or mean ± SD
Age (years)	60.5 ± 8.1
*Age categories (%)*	
48.0–55.0	31.9
55.1–64.9	35.2
65.0–84.0	32.9
Female (%)	41.3
Israeli-born (%)	69.9
Arab (%)	18.9
Married/cohabited (%)	81.6
Socioeconomic position	5.8 ± 2.2
Never smokers (%)	49.2
Education	
< 12 years (%)	19.9
12 years (%)	34.4
> 12 years (%)	45.7
Diabetes duration	2.7 ± 1.0
Anti-diabetic medications^b^ (%)	73.0
Comorbidities^a^ (%)	29.8
*Process indicators- performance in 2017 (%)*	
HbA1c	92.9
LDL-cholesterol	93.6
Blood pressure	91.6
Serum creatinine	94.1
Urinary protein	91.6
Eye clinic visit	52.6
Influenza vaccination	41.8
*Process indicators- composite score in 2017 (%)*	
0–4	10.7
5	25.0
6	41.8
7	22.4
*Intermediate-outcome indicators- controlled in 2017 (%)*	
HbA1c (≤ 7%/ ≤ 8%)*	71.9
LDL-cholesterol ≤ 100 mg/dL	49.0
Blood pressure ≤ 140/90 mmHg	76.5

LDL-cholesterol: low density lipoprotein cholesterol

^a^Comorbidities included: cardiac disease, retinopathy, visual loss, end stage renal disease or lower limb amputation based on diagnosis and procedures

^b^Oral hypoglycemic medications (none was treated with insulin)

*HbA1c ≤ 7% for patients aged ≤ 74 years and HbA1c ≤ 8% for patients aged ≥ 75 years. Among patients who were treated with anti-diabetic medications 75.0% had a controlled level of HbA1c (≤ 7%/ ≤ 8%)

### Physical and mental health

Mean scores of the physical and mental health scales were 43.9 (SD:10.5) and 50.1 (SD:9.3), respectively. Table 2 presents the associations between socio-demographic and clinical variables with physical and mental health scores. Female sex and younger age were negatively associated with physical health score (β_female_ = − 4.96, 95% CI [− 7.00, − 2.91] and β_48–55years_ = − 3.26, 95% CI [− 5.88, − 0.64] vs. 65–84 years). Moreover, lower level of education was associated with worse physical health score (β_<12 years_ = − 7.73, and β_12 years_ = − 2.85 vs. > 12 years). Regarding clinical characteristics, presence of comorbidities (β = − 3.82, 95% CI [− 6.10, − 1.53]) and performance of at least six of the process indicators (β_6 indicators_ = − 5.05 and β_7 indicators_ = − 5.79) were associated with worse physical health.

**Table 2:** Associations between socio-demographic and clinical factors with physical and mental health (n = 392)

	Categories	Physical health^a^				Mental health^a^			
		Model 1		Model 2		Model 1		Model 2	
		β	95% CI	β	95% CI	β	95% CI	β	95% CI
Gender	Female versus male	− 3.74**	− 5.77, − 1.71	− 4.96**	− 7.00, − 2.91	− 2.05*	− 3.89, − 0.20	− 2.73*	− 4.61,− 0.84
Age	48.0–55.0 y	− 1.17	− 3.70, 1.36	− 3.26*	− 5.88, − 0.64	1.74	− 0.56, 4.04	0.36	− 2.06, 2.78
	55.1–64.9 y	0.29	− 2.16, 2.74	− 1.14	− 3.63, 1.35	0.92	− 1.30, 3.14	− 0.05	− 2.35, 2.25
	65.0–84.0 y	Ref		Ref		Ref		Ref	
Ethnicity	Arabs versus Jews	0.31	− 2.49, 3.12	0.39	− 2.35, 3.12	1.39	− 1.16, 3.93	1.35	− 1.18, 3.87
Education	< 12 y	− 8.07**	− 10.93, − 5.21	− 7.73**	− 10.52, − 4.94	− 6.23**	− 8.83, − 3.64	− 5.96**	− 8.54, − 3.38
	12 y	− 3.38*	− 5.62, − 1.14	− 2.85*	− 5.04, − 0.66	− 2.83*	− 4.87, − 0.80	− 2.54*	− 4.56, − 0.51
	> 12y	Ref		Ref		Ref		Ref	
Comorbidities^b^	Yes versus no	–	–	− 3.82**	− 6.10, − 1.53	–	–	− 2.11^#^	− 4.21, 0.00
Composite score	0–4	–	–	Ref		–	–	Ref	
	5	–	–	− 3.06	− 6.62, 0.50	–	–	− 2.35	− 5.64, 0.94
	6	–	–	− 5.05*	− 8.39, − 1.71	–	–	− 2.57	− 5.66, 0.51
	7	–	–	− 5.79*	− 9.47, − 2.11	–	–	− 4.59*	− 7.99, − 1.19

^a^T-score of the 4 items for physical or mental health from PROMIS-10. Multivariable generalized linear models, model 1 included socio-demographic variables and model 2 included also the clinical variables

^b^Comorbidities included: cardiac disease, retinopathy, visual loss, end stage renal disease or lower limb amputation based on diagnosis and procedures

*P-value < 0.05; ** P-value < 0.001; ^#^ P-value = 0.05

While age was not associated with mental health score, female sex was associated with worse mental health (β = − 2.73, 95% CI [− 4.61, − 0.84]). Lower level of education was associated with worse mental health score (β_<12 years_ = − 5.96, 95% CI [− 8.54, − 3.38] and β_12 years_ = − 2.54, 95% CI [− 4.56, − 0.51] vs. > 12 years). Regarding clinical characteristics, presence of comorbidities (β = − 2.11, 95% CI [− 4.21, 0.00], P-value = 0.05) and performance of all seven process indicators (β = − 4.59, 95% CI [− 7.99, − 1.19]) were negatively associated with mental health. It is worth mentioning that in a separate PROMIS-10 question of rating their general health, a higher proportion of those who achieved 6–7 process indicators rated their general health as poor or fair, compared to those who achieved 0–5 indicators (34.5% vs. 24.3%, respectively, P-value = 0.023).

### Diabetes-related distress

Among the participants, 22.2% had a high level of diabetes-related distress (PAID ≥ 40). Diabetes-related distress correlated negatively and significantly (p < 0.01) with physical and mental health (Spearman-r_=_ − 0.51 and − 0.46, respectively, Additional file 1: Table S1).

Table 3 presents the associations between the independent variables and PAID. Women and younger patients were more likely to experience high diabetes-related distress (OR _Female_ = 1.84, OR_48.0–55.0years_ = 4.41, and OR_55.1–64.9years_ = 2.81 vs. 65–84 years). Patients with a low education level were more likely to experience high diabetes-related distress (OR_<12years_ = 5.27 and OR_12years_ = 2.20 vs. > 12 years). Patients who achieved 5–7 process indicators were more likely (ORs = 3.07–3.60) to experience high diabetes-related distress compared to patients who achieved only 0–4 indicators.

**Table 3:** Adjusted odds ratios (ORs) of high diabetes-related distress^a^, multivariable logistic regression (n = 392)

	Categories	Model 1		Model 2	
		OR	95% CI	OR	95% CI
Gender	Female versus male	1.56	0.94, 2.60	1.84*	1.07, 3.15
Age	48.0–55.0 y	3.28**	1.65, 6.52	4.41**	2.09, 9.29
	55.1–64.9 y	2.42*	1.22, 4.78	2.81*	1.38, 5.74
	65.0–84.0 y	Ref		Ref	
Ethnicity	Arabs versus Jews	0.60	0.30, 1.19	0.58	0.29, 1.16
Education	< 12 y	5.16**	2.53, 10.53	5.27**	2.55, 10.91
	12 y	2.33*	1.29, 4.23	2.20*	1.21, 4.03
	> 12y	Ref		Ref	
Comorbidities^b^	Yes versus no	–	–	1.47	0.80, 2.72
Composite score	0–4	–	–	Ref	
	5	–	–	3.07*	1.02, 9.27
	6	–	–	3.09*	1.07, 8.98
	7	–	–	3.60*	1.15, 11.28

^a^PAID: Problem Areas in Diabetes, a score ≥ 40 indicates high diabetes-related distress

^b^Comorbidities included: cardiac disease, retinopathy, visual loss, end stage renal disease and lower limb amputation based on diagnosis and procedures

*P-value < 0.05; **P-value < 0.001

### Sexual dysfunction, self-management of diabetes, integrated care, costs of care, and shared decision making

Of the participants, 45% reported that cost of anti-diabetic medication presented a problem for them (Additional file 1: Fig. S1). Approximately 30% of the participants reported that they suffered from sexual dysfunction, 29% reported lack of integrated care and 30% being unconfident in their management of the disease. Almost 45% reported a lack of shared decision-making (Additional file 1: Fig. S1).

Table 4 presents the associations between the independent variables, including the demographic and clinical variables, and the PROMs. Arabs and those with low education level (≤ 12 years) were independently more likely (ORs≈2) to face problems with anti-diabetic medications’ costs compared to Jews and those with high education, respectively. Men, Arabs, and patients with low education level were more likely to report on sexual dysfunction. Low reported self-management ability was significantly associated with female sex, young age, and lower level of education (ORs = 2–3). Lack of shared decision-making was more likely to concern women, older patients, and Jews. A substantial number of patients answered ‘irrelevant’ in the items, lack of integrated care under one roof (24.0%) and shared decision-making (25.5%). Therefore, we repeated the analyses using multinomial logistic regressions with ‘irrelevant’ as a separate category. These analyses yielded similar results. Comorbidities and composite score were not associated significantly with these issues.

**Table 4:** Adjusted odds ratios (ORs) of problems (yes vs. no), multivariable logistic regressions (n = 392)

Model 1^a^		Anti-diabetic medications’ cost		Sexual dysfunction		Lack of integrated care under one roof		Unconfident in self-management		Lack of shared decision-making	
		OR	95% CI	OR	95% CI	OR	95% CI	OR	95% CI	OR	95% CI
Gender	Female versus male	1.48	0.96, 2.28	0.40**	0.24, 0.68	1.21	0.72, 2.04	2.03*	1.28, 3.20	1.56*	1.01, 2.41
Age	48.0–55.0 y	1.13	0.65, 1.94	1.42	0.77, 2.61	3.19**	1.59,6.42	2.46*	1.36, 4.47	0.49*	0.29, 0.85
	55.1–64.9 y	1.08	0.64, 1.82	1.18	0.64, 2.16	2.55*	1.28, 5.09	1.79^#^	1.00, 3.22	0.43*	0.25, 0.72
	65.0–84.0 y	Ref		Ref		Ref		Ref		Ref	
Ethnicity	Arabs versus Jews	1.83*	1.01, 3.32	3.67**	2.00, 6.76	10.11**	5.23,19.52	0.82	0.44, 1.52	0.53*	0.30, 0.94
Education	< 12 y	2.34**	1.28, 4.29	2.90**	1.52, 5.53	1.58	0.76, 3.28	2.99**	1.58, 5.67	0.71	0.39, 1.28
	12 y	1.99**	1.23, 3.20	1.44	0.83, 2.48	1.36	0.76, 2.42	1.67^#^	0.99, 2.80	1.21	0.75, 1.94
	> 12y	Ref		Ref		Ref		Ref		Ref	

Except for medication costs (n = 371); excluding irrelevant

^a^Model 1 and model 2 revealed similar results, comorbidities and composite score were not associated significantly with the selected items. * P-value < 0.05; ** P-value < 0.001. ^#^ P-value = 0.05

No significant associations were found between PROMs (diabetes-related distress, physical and mental health as well as the additional items) and levels of HbA1c, blood pressure, or LDL-cholesterol, as continuous variables or intermediate-outcome indicators (data not shown).

## Discussion

In this cross-sectional study, we assessed the potential utility of PROMs in type 2 diabetes care for the first time in Israel. We investigated the associations between PROMs and numerous clinical quality indicators. While adherence with traditional process indicators (e.g., HbA1c tests) was associated with worse general health and high diabetes-related distress, the measured levels of HbA1c, LDL-cholesterol, or blood pressure were not associated with PROMs, suggesting that PROMs capture additional dimensions, i.e. outcomes, not reflected in clinical indicators.

Our study found, in agreement with previous studies, that women, young patients, and those with low education reported worse general health [19, 42–46], high diabetes-related distress [37, 47–53] and low confidence in diabetes self-management [54, 55]. Physiological and lifestyle factors, including diet and physical activity, may contribute to the gender and educational differences [46, 55, 56]. In the current study, young patients reported worse physical health compared to patients aged > 65 years. It may well be that older patients show higher levels of acceptance of diabetes-related restrictions and attribute those to aging, unlike younger individuals. Indeed, a previous study indicated that the proportion of patients who reported not being limited by their diabetes increased with age [19]. It was speculated that self-management is particularly difficult for young patients, who are busy in their careers [55, 57].

Interestingly, our study showed that performance of all process clinical indicators was associated with poor physical and mental health. Previous studies showed different results; one study showed that neither physical nor mental health was associated with performance of process indicators [27], but another study showed that performance of process indicators was associated with better mental health [58]. Given the cross-sectional nature of this study, we cannot establish the direction of associations between indicators’ performance and poor general health. It is worth mentioning two potential explanations. First, it may well be that patients characterized with high level of anxiety evaluate their health status as poor and also tend to perform all the process indicators. Second, the presence of comorbidities could explain part of the association under consideration. Indeed, we have controlled for comorbidities, yet the adjustment may have been insufficient as we did not consider neither illness severity nor the number of comorbidities. In addition, we found that patients who achieved most (5–7) of the process indicators were more likely to have high diabetes-related distress compared to those who achieved less. The aforementioned potential explanations, anxiety and presence of comorbidities, may partly explain the association between indicators’ performance and diabetes-related distress. Moreover, possibly increased medical testing, per se*,* increases patient’s diabetes-related distress. Indeed, previous studies indicated that a higher frequency of self-monitoring of blood glucose was associated with higher levels of distress and worries [52, 59]. Thus, health providers should be aware that adherence to clinical indicators, may not indicate better health status or less diabetes-related distress among patients recently diagnosed with type 2 diabetes.

In our study, no significant association was detected between control of HbA1c, LDL-cholesterol, or blood pressure, and general health. This is consistent with previous studies [60–64] that showed no association between general health and level of HbA1c. Also, our study suggests no association between control of HbA1c and diabetes-related distress. This is in line with previous studies [47, 49, 65] that focused, as this study does, on recently diagnosed patients. While others have shown that poor glycemic control was associated with high diabetes-related distress [37, 51, 52, 66–68], most of these studies included patients with a long-standing diabetes. Thus it would be interesting to test whether the associations are modified by diabetes duration.

In this study, costs of medications were considered a problem for almost half of the participants. In Israel, there is a national health insurance law that subsidizes only some of the medications with relatively low co-payments. Several factors may explain this finding, including the purchase of medications not covered by health insurance; the presence of comorbidities; low socioeconomic position; and, since the patients were recently diagnosed with type 2 diabetes, the fact that medication cost was a new, additional financial burden. The issue of medication cost was also highlighted in our previous qualitative study, where patients with type 2 diabetes expressed concerns about the financial burden of medications, stating: “There’s a new medication that’s effective. But the packet costs 250 shekels [73 USD]. What about the low-paid workers or the elderly who live off of their pensions, how could they pay for that? They cannot.” [34]. Another Israeli study showed that 10% of patients with diabetes were non-adherent with pharmacotherapy due to cost; and this behavior was associated with low socioeconomic position, unemployment, and lack of physician explanation about the prescribed medication [69].

Our findings showed that almost a third of the participants reported that they suffered from sexual dysfunction. In a multinational study, sex life was found to be the fourth most important issue out of 19 diabetes-specific domains [42]. Nevertheless, the current standard questionnaires do not include information on sexual dysfunction. Moreover, sexual dysfunction is often overlooked by health practitioners: 63% of patients with diabetes reported that their physicians had never addressed their sexual problems [70]. PROMs could be a useful tool to identify patients with sexual dysfunction and to follow them up.

Integrating PROMs into the routine clinical practice is a challenge[71]. Health providers need effective approaches to use PROMs without disrupting traditional care. Multidisciplinary healthcare teams are required for diabetes care (e.g., primary care physicians, and mental health professionals) and some aspects are more relevant to some professionals than others. Thus we recommend specifying who will address each of the PROMs. Moreover, technology (e.g. tablets in the clinics) seems to play a central role in the routine collection and use of PROMs in clinics [12]. PROMs in diabetes care should be monitored regularly, ICHOM has suggested to collect PROMs at baseline and annually [20] and we suggest considering the collection of PROMs also surrounding a change in treatment plan and when a complication is diagnosed.

Limitations of this study should be acknowledged. First, this study used a cross-sectional design, which allows assessing associations but not causal relationships and can’t determine the temporal relationship among the variables examined. A longitudinal study is needed to gain an in-depth understanding of the associations. Second, the clinical indicators were assessed in 2017 and the PROMs were assessed in 2019. Third, 4-items were added by the authors (medication costs, sexual dysfunction, shared decision-making, and integrated care under one roof). Although these items were validated by experts, almost 25% of the participants have answered "irrelevant" for the last two items. Notably, sensitivity analyses were conducted using multinomial logistic regressions with “irrelevant” as a separate category, and the results were similar to those achieved from the main analyses. Lastly, the study focused on patients relatively recently diagnosed with type 2 diabetes, and the conclusions are limited to this group.

However, our study also has some important strengths. First, the results suggest that PROMs are applicable for routine measurement of diabetes quality care; the examined questionnaires are free for use in clinical practice, with acceptable length and are easily scored. Second, the examined PROMs were based on incorporating both the standard set recommended by ICHOM and aspects that are somewhat neglected in the existing PROMs, such as sexual dysfunction and financial burden, which were found valuable for patients with type 2 diabetes in our previous qualitative study [34]. Third, we used well-validated questionnaires and the combination of generic and diabetes-specific PROMs provided a comprehensive assessment. Lastly, the study estimated the associations between PROMs and comprehensive clinical quality indicators that commonly used in diabetes care, enabling the estimation of the added value of PROMs.

## Conclusions and policy implications

Our study suggests that PROMs capture important information on patient health status and quality of type 2 diabetes care that are not reflected in the examined clinical quality indicators. PROMs are powerful tools, with the potential to both expand the evaluation of the outcomes of diabetes care and promote person-centered care.

Managing diabetes is a demanding task, requiring self-management (e.g., healthy lifestyle, blood glucose monitoring and medication adjustment) and individualized multi-disciplinary medical and psychosocial care in order to prevent complications and maintain a satisfactory quality of life. Active clinical use of PROMs and responding to the collected PROMs may improve multiple aspects of diabetes quality care. PROMs could aid health providers in identifying patient’s needs, responsiveness to interventions, and enable outcome monitoring. PROMs promote patient-provider collaboration, which in turn may improve patient’s adherence and quality of life. Aggregate PROMs of the primary clinic could assist medical directors in identifying at-risk groups and their needs, enhancing team members education and allocating resources accordingly.

In conclusion, this study suggests that PROMs and clinical quality indicators reflect different aspects of the quality of diabetes care and both should be considered to promote person-centered care, to improve the quality of diabetes care, and to achieve a comprehensive evaluation of diabetes quality care. Thus, we recommend that policy-makers within the Ministry of Health and health maintenance organizations promote the implementation of PROMs in type 2 diabetes care and allocate resources for further evaluation of how to implement it most efficiently, which potentially could be within the Israeli National Program for Quality Indicators in Community Healthcare.

### Supplementary Information

**Additional file 1**. **Table S1.** Correlations (Spearman) between PROMs, socio-demographics and quality indicators, (n = 392). **Figure S1.** Distribution of facing problems in selected items, (n = 392).

## Data Availability

The datasets analyzed during the current study are not publicly available due to ethical restrictions on sharing de-identified data sets, participants had not provided informed consent on sharing data publicly. The data that support the findings of this study are available upon reasonable request and with the approval of the Institutional Review Board of Maccabi Healthcare Services.

## Abbreviations

PROMs: Patient-Reported Outcome Measures
PAID: Problem Areas in Diabetes
ICHOM: International Consortium for Health Outcomes Measurement
GPH: Global physical health
GMH: Global mental health
