# Supplementary material for: Implications of Patient-Reported Outcome Measures among patients with recently diagnosed type 2 diabetes
Source: Isr J Health Policy Res. 2024 Jan 31;13:6. doi: 10.1186/s13584-024-00592-1 (PMC10829200; doi:10.1186/s13584-024-00592-1)
Supplement: Supplementary file 1 — Additional file 1. Table S1. Correlations (Spearman) between PROMs, socio-demographics and quality indicators, (n = 392). Figure S1. Distribution of facing problems in selected items, (n = 392). [file 13584_2024_592_MOESM1_ESM.docx]

# **Additional file**

**Table S1.** Correlations (Spearman) between PROMs, socio-demographics and quality indicators, (n=392).

| Spearman | GMH | GPH | Age | Education | SEP | HbA1c | LDL | SBP | Composite |
| --- | --- | --- | --- | --- | --- | --- | --- | --- | --- |
| PAID | **-0.46**** | **-0.51**** | **-0.21**** | **-0.24**** | -0.05 | ***0.115** | 0.08 | -0.27 | 0.09 |
| GMH | 1 | ****0.67** | -0.09 | **0.28**** | **0.10*** | -0.029 | 0.04 | -0.005 | **-0.14**** |
| GPH |  | 1 | 0.03 | **0.34**** | **0.18**** | -0.011 | 0.08 | 0.04 | **-0.16**** |

PROMs: Patient-Reported Outcome Measures and included; PAID: Problem Areas in Diabetes, GPH: Global Physical Health, GMH: Global Mental Health. SEP: socioeconomic position, HbA1C: glycated hemoglobin, LDL: low-density lipoprotein cholesterol, SBP: Systolic blood pressure, Composite: composite process score (0-7). Age and education in years. * p<0.05, ** p<0.01.

**Figure S1**. Distribution of facing problems in selected items, (n=392).
